# Supplementary material for: The Vacuolar Morphogenesis Protein Vam6-Like Protein Vlp1 Is Required for Pathogenicity of Cryptococcus neoformans
Source: J Fungi (Basel). 2021 May 27;7(6):418. doi: 10.3390/jof7060418 (PMC8228526; doi:10.3390/jof7060418)
Supplement: Supplementary file 1 [file jof-07-00418-s001.zip › jof-1219801-supplementary.pdf]

Journal of *Fungi*

MDPI

Supplementary Material

The Vacuolar Morphogenesis Protein Vam6-Like Protein Vlp1 Is Required for Pathogenicity of *Cryptococcus neoformans*

Cheng-Li Fan [1] and Tong-Bao Liu [2,3,*]

[1] College of Animal Science and Technology, Southwest University, Chongqing 400715, China; victoriafan@ swu.edu.cn
[2] State Key Laboratory of Silkworm Genome Biology, Southwest University, Chongqing 400715, China
[3] Medical Research Institute, Southwest University, Chongqing 400715, China
* Correspondence: tongbaoliu@swu.edu.cn; Tel.: +86-23-6825-1088

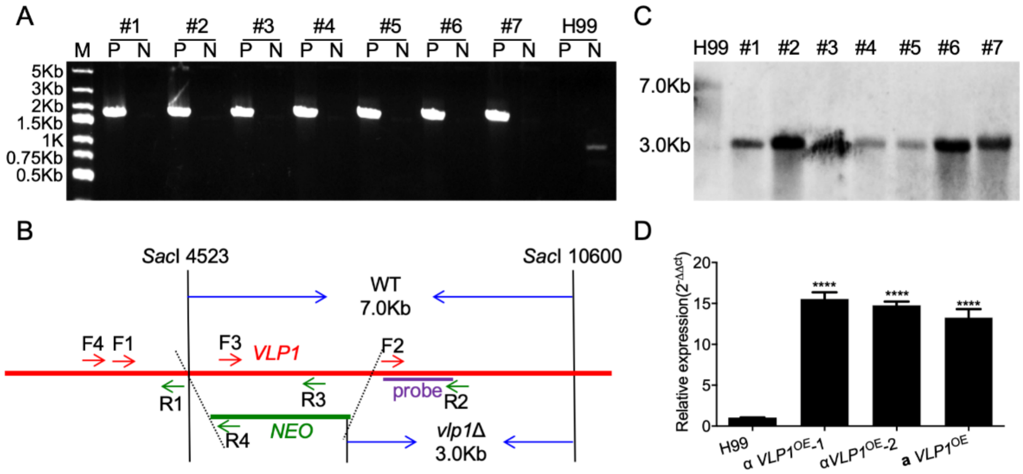

Figure S1. Generation of *vlp1*Δ mutants and *VLP1* overexpressed strains. (**A**) PCR verification of the G418 resistant transformants. #1-7: Seven G418 resistant transformants; P: positive primers (TL333/59, F4/R4 in 2B); N: negative primers (TL331/332, F3/R3 in 2B). (**B**) Restriction enzyme used for genomic DNA digestion and DNA probe for Southern blot. The probe was synthesized using the downstream flanking sequence of the *VLP1* gene as a template. The wild-type strain H99 produced a 7.0 Kb band, while the *vlp1*Δ mutants produced a 3.0 Kb band. (**C**) Southern blot analysis of *VLP1* deletion mutants. The *Sac*I-digested genomic DNAs of each cryptococcal strains were fractionated and hybridized with a probe synthesized using the *VLP1* downstream flanking sequence as a template, as shown in Figure S1B. The wild-type strain H99 generate a 7.0 Kb band while the *vlp1*Δ mutants generate a 3.0 Kb band. (**D**) The overexpression of the *VLP1* gene was verified by relative quantitative reverse transcription PCR. The expression level of the *VLP1* gene of the wild-type strain, when grown on YPD medium, was set as 1. ****, $p < 0.0001$.

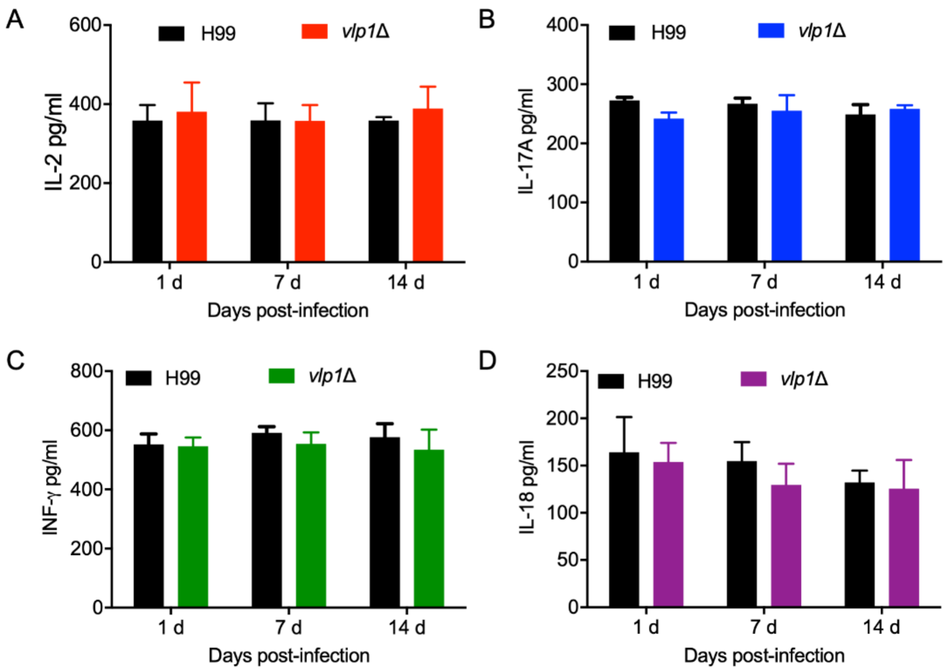

Figure S2. Serum cytokines analysis of the mice infected by *vlp1*Δ mutants. IL-2 (**A**), IL-17A (**B**), IFN-$\boldsymbol{\gamma}$ (**C**), and IL-18 (**D**) were measured by ELISA at 1, 7,14 dpi. There was no difference in Serum cytokines production in mice infected with wild-type strains and *vlp1*Δ mutants.
